# Rapid exploration of the epitope coverage produced by an Ebola survivor to guide the discovery of therapeutic antibody cocktails

**DOI:** 10.1093/abt/tbaa016

**Published:** 2020-08-01

**Authors:** Tom Z Yuan, Ana G Lujan Hernandez, Erica Keane, Qiang Liu, Fumiko Axelrod, Shweta Kailasan, Madeleine Noonan-Shueh, Mohammad Javad Aman, Aaron K Sato, Yasmina N Abdiche

**Affiliations:** Twist Biopharma, Twist Bioscience, South San Francisco, CA 94080, USA; Twist Biopharma, Twist Bioscience, South San Francisco, CA 94080, USA; Twist Biopharma, Twist Bioscience, South San Francisco, CA 94080, USA; Twist Biopharma, Twist Bioscience, South San Francisco, CA 94080, USA; Twist Biopharma, Twist Bioscience, South San Francisco, CA 94080, USA; Integrated Biotherapeutics Inc., Rockville, MD 20850, USA; Integrated Biotherapeutics Inc., Rockville, MD 20850, USA; Integrated Biotherapeutics Inc., Rockville, MD 20850, USA; Twist Biopharma, Twist Bioscience, South San Francisco, CA 94080, USA; ImmunoPrecise Antibodies Ltd., Fargo, ND 58104, USA

**Keywords:** epitope binning, antibody therapeutics, Ebola virus, surface plasmon resonance, infectious disease, neutralizing antibodies, synthetic biology

## Abstract

**Background:**

Development of successful neutralizing antibodies is dependent upon broad epitope coverage to increase the likelihood of achieving therapeutic function. Recent advances in synthetic biology have allowed us to conduct an epitope binning study on a large panel of antibodies identified to bind to Ebola virus glycoprotein with only published sequences.

**Methods and Results:**

A rapid, first-pass epitope binning experiment revealed seven distinct epitope families that overlapped with known structural epitopes from the literature. A focused set of antibodies was selected from representative clones per bin to guide a second-pass binning that revealed previously unassigned epitopes, confirmed epitopes known to be associated with neutralizing antibodies, and demonstrated asymmetric blocking of EBOV GP from allosteric effectors reported from literature.

**Conclusions:**

Critically, this workflow allows us to probe the epitope landscape of EBOV GP without any prior structural knowledge of the antigen or structural benchmark clones. Incorporating epitope binning on hundreds of antibodies during early stage antibody characterization ensures access to a library’s full epitope coverage, aids in the identification of high quality reagents within the library that recapitulate this diversity for use in other studies, and ultimately enables the rational development of therapeutic cocktails that take advantage of multiple mechanisms of action such as cooperative synergistic effects to enhance neutralization function and minimize the risk of mutagenic escape. The use of high-throughput epitope binning during new outbreaks such as the current COVID-19 pandemic is particularly useful in accelerating timelines due to the large amount of information gained in a single experiment.

Statement of Significance: Starting with only published antibody sequences and soluble Ebola virus glycoprotein, high-throughput surface plasmon resonance is employed to rapidly screen and epitope bin hundreds of antibodies to fully characterize the epitope coverage of antibodies identified from a convalescent donor.

## INTRODUCTION

Emerging infectious diseases with epidemic potential, such as severe acute respiratory syndrome (SARS), Ebola virus (EBOV) disease, and the novel coronavirus disease 2019 (COVID-19) underscore the increasing need for the rapid development of vaccines and post-exposure therapies. Antibody therapeutics that target the EBOV glycoprotein (EBOV GP), one of several gene products that interact with host cells during EBOV pathogenesis [1]**,** have been shown to be effective, particularly when administered as cocktails of monoclonal antibodies (for simplicity, herein referred to as ‘antibodies’) that achieve broad epitope coverage of the target antigen enabling potent, long-lasting neutralization by complementary mechanisms of actions [2–9]. Cocktail approaches mimic the natural polyclonal immune response and rely on combining antibodies targeting different epitopes to unlock synergistic effects that can boost *in vivo* protection orders of magnitude compared with monotherapy, as reported in the neutralization of various biological toxins [10]. Furthermore, since viral antigens have evolved a remarkable propensity to mutate rapidly as a strategy to defy human immune responses, antibody cocktails with broad epitope coverage lower the risk of mutagenic escape, which will otherwise render antibodies ineffective, as observed in nonhuman primates following treatment with a cocktail comprised of antibodies targeting highly similar epitopes on the EBOV GP [11]**.**

Although characterizing the antigenic surface of viral glycoproteins is advantageous in developing therapies, detailed information on their structure and the roles of particular binding epitopes in protection are often lacking, which poses a critical bottleneck in responding to new outbreaks or viral isolates. Furthermore, discrete epitopes of the viral antigen may play distinct mechanistic roles that are unknown, cooperative, [3, 7] and have varying levels of risk for mutagenic escape [12]. These factors confound the ability to design effective cocktail therapeutics.

Epitope binning is a useful empirical method for organizing antibodies into epitope families by assessing the blocking relationships that emerge from a pairwise and combinatorial competition of antibodies against their specific target antigen [13]. However, when studying large panels of antibodies comprising hundreds of clones, exploring an exhaustive pairwise competition matrix of the entire set by standard technologies such as FACS, ELISA, and label-free biosensors is tedious and resource-intensive, so for practical reasons, the scope of these assays is often limited to the blockade against a small set of benchmark ‘reagent’ antibodies of known specificity or function. Antibodies that are binned in competition with a handful of controls constitute a ‘few-on-many’ approach and rely upon the existence of such standards. In contrast, high-throughput epitope binning assays that expand the number of pairwise permutations that can be addressed in a single experiment not only provide practical advantages of speed and minimal sample consumption, but also provide exquisite resolution revealing small differences in epitope specificity and nuanced binding modes such as allosteric modulation that may be relevant for functional activity [14–16]. Additionally, such assays are self-referencing and do not require the use of controls, so are universally applicable to any antibody library as soon as sufficient protein is expressed and provided that the target antigen is available [17]. Improvements in the throughput of label-free biosensor technologies such as Octet-HTX (ForteBio), IBIS-MX96 (IBIS Technologies), and LSA (Carterra) enable the use of epitope binning as a high-throughput screening process rather than being reserved for small panels of antibodies [13, 16]. Previous studies have employed this method to determine fine epitope differences among antibodies to human ﻿progranulin [16] and programmed cell death protein 1 [18].

**Figure 1:**
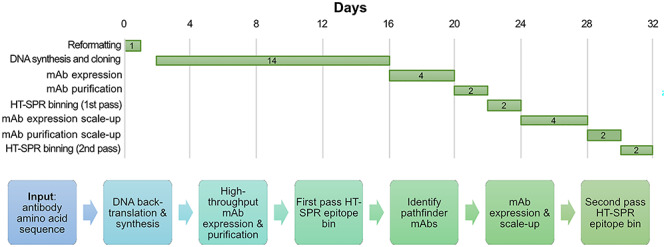
Timeline and workflow for the production and analysis of a large panel of anti-EBOV GP antibodies identified from a human survivor of Ebola virus infection [2] to enable a high-throughput surface plasmon resonance (HT-SPR) binning study. See Materials and Methods for details.

Understanding the epitope coverage produced by the human immune repertoire in response to authentic EBOV infection can guide the design of antibody cocktails for all viral infectious diseases. In this study, we build on the work of Bornholdt et al***.*** in characterizing the antibodies recovered from the B cell response of a convalescent donor from the 2014 Zaire EBOV epidemic [2]. We describe a high-throughput surface plasmon resonance (HT-SPR), minimally consumptive epitope binning workflow that recapitulates the epitope bins of this human-sourced antibody library within a 4 week timeline without the use of any antibody controls or antigen structural information (Fig. 1). Synthetic biology advances enabled the rapid, high-throughput DNA synthesis, expression, and purification of 321 antibodies with only the variable domains’ amino acid sequences as an input. The EBOV GP target was chosen as it is well-studied, biologically relevant, and available in recombinant purified form commercially. This set of antibodies was chosen due to the availability of in-depth characterization reported in the literature, allowing us to benchmark our binning assignments to published bins assigned by FACS. Following a nonoptimized, ‘first pass’ HT-SPR binning that revealed the epitope landscape including rare epitopes, representative antibodies were selected from each bin. These ‘pathfinder’ antibodies were further investigated in an independently conducted ‘second pass’ binning along with benchmark anti-EBOV GP antibodies supplemented with orthogonal data such as antibody sequence, *in vitro* cell-based neutralization of live virus, and *in vivo* survival to lethal challenge in mouse models to provide a comprehensive analysis that can aid in the rational design of therapeutic cocktails.

## MATERIALS AND METHODS

### Reformatting, expression, and purification of anti-EBOV GP antibodies

Variable heavy and light domains of anti-EBOV GP antibodies were sourced from Bornholdt et al. [2] and reformatted to IgG1 and submitted as a clonal gene order for DNA back-translation, synthesis, and cloning into mammalian expression vector pTwist CMV BG WPRE Neo through the Twist Bioscience eCommerce portal. Light chain variable domains were reformatted into kappa and lambda frameworks accordingly. Clonal genes were delivered as purified plasmid DNA ready for transient transfection in HEK Expi293 (Thermo Scientific). Cultures at a volume of 1.2 mL were grown to 4 days, harvested, and purified using Protein A coated magnetic beads on KingFisher Flex. CE-SDS was used to determine antibody purity and confirm molecular weight. For the second pass binning, a smaller set (24 total) of antibodies was selected for transient transfection in 30 mL cultures using the same expression system. Cultures were grown for 4 days, harvested, and purified with Phynexus Protein A resin tips on Hamilton Star automated liquid-handling systems. Purified antibodies were concentrated using Amicon, 30 kDa, spin filters. All antibodies were eluted with 50 mM sodium acetate followed by 140 mM HEPES neutralization (final 50 mM sodium acetate, 140 mM HEPES pH 6.0).

### Structural benchmark clones and antigens

Thirteen antibodies with defined structural epitopes from the literature were supplied in purified form (Integrated Biotherapeutics); ADI-15878 (Protein Data Base (PDB) ID 6DZN), ADI-15946 (PDB 6DZN), ADI-16061 [19], c13C6 [20], c2G4-N [20], c4G7-N [20], CA45 (PDB 6EAY), FVM-04 [7], FVM-09 [7], h4B8 [21], KZ52 (PDB 3CSY), mAb100 (PDB 5FHC), and mAb114 (PDB 5FHC). Integrated Biotherapeutics also supplied the antigen, EBOV GP, a trimer of the GP1 + GP2 heterodimer (69.4 kDa; AA33–637 of NP_066246.1; isolated from Ebola virus, Mayinga strain) lacking the transmembrane domain with an assembled molecular mass of ~208 kDa produced in *Drosophila* S2 cells as described previously [6]. The EBOV GP sample used in all epitope binning experiments appeared to be homogeneous in analytical size exclusion chromatography while migrating at an apparent molecular mass of ~386 kDa, indicating substantial glycosylation (Supplementary Fig. S1).

### HT-SPR epitope binning

Epitope binning was performed in a premix format using a Carterra LSA SPR biosensor equipped with a HC30M chip at 25 °C in HBS-TE (10 mM HEPES pH 7.4, 150 mM NaCl, 3 mM EDTA, 0.05% Tween-20). In the first pass binning, antibodies were diluted 1:50 from the original expression yield **(**Supplementary Fig. S2**)** and amine-coupled to the sensor chip by EDC/NHS activation, followed by ethanolamine HCl quenching. The concentrations of EBOV GP and antibody used in premixes were calculated in terms of binding sites, with antibody in molar excess. An initial binding test and regeneration scout suggested that robust and reproducible binding to most ligands was achieved using 80 nM EBOV GP with regeneration in 75 mM phosphoric acid. Ligands with good binding responses and expression yields were used as premixed analyte in first-pass bin and diluted 1:5 for the original expression yield, with EBOV GP fixed at 80 nM (final). Each premix sample was injected over the immobilized ligands to determine blocking, partial blocking or nonblocking. In the second pass binning, antibody concentrations were normalized, and premixes were assembled with 50 nM EBOV GP and 300 nM antibody.

Data were analyzed in Carterra’s Epitope Tool software. Briefly, blocking assignments were determined relative to the binding responses for EBOV GP alone (normalized to 1); premixes giving binding responses less than 0.5 were determined to be blocking, 0.5–0.7 were intermediate blocking, while above 0.7 were not blocking. Heat maps representing the competition results were generated where red, yellow, and green cells represent blocked, intermediate, and not blocked analyte/ligand pairs, respectively. White cells represent unaddressed pairs in the assay. For more detail, see **Supplementary Materials and Methods**.

## RESULTS

### High throughput epitope binning of EBOV antibodies revealed seven distinct epitope ‘communities’ without the use of structural benchmark antibodies

A nonoptimized ‘first pass’ HT-SPR epitope binning study was performed on the antibody library expressed at small scale (1.2 mL culture) by coupling the antibodies as a 384-ligand array. The 321 antibodies produced were expressed in various yield (from 2–64 μg, with mean of 17 μg) and supplied in 90 μL/antibody (corresponding to 20–710 μg/ml, with mean of 190 μg/ml)—see Supplementary Fig. S2. They were coupled at a 50-fold dilution of the supplied stock (final 0.4–14 μg/ml, with mean of 4 μg/ml). While no attempt was made to normalize their concentrations during the coupling step, this ‘batch dilution’ method produced active ligands with robust antigen-binding responses for the majority of antibodies, even those coupled at very low concentrations.

**Figure 2:**
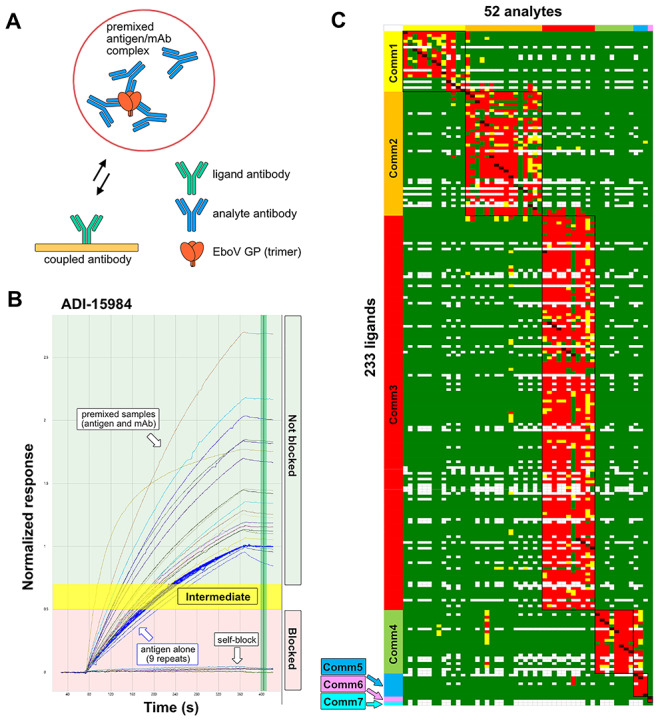
First pass HT-SPR epitope binning of anti-EBOV GP antibodies. (A) The cartoon illustrates the premix assay format used in the binning assay, where a molar excess of antibody (in binding sites) is combined with the EBOV GP trimer complex of GP1 + GP2 heterodimer in solution to form a premixed sample that is injected (as analyte) over an array of coupled antibodies (as ligands). (B) Example sensorgram overlay plot displaying the normalized binding responses detected on ligand ADI-15984 of EBOV GP alone (blue curves, reaching a normalized response of 1), EBOV GP premixed with ADI-15984 giving a full self-block (baseline response), and EBOV GP premixed with various antibodies that did not block ADI-15984 (giving responses at or above that of antigen alone). Vertical green line marks timepoint at which each interaction is assigned as blocking (red area), intermediate blocking (yellow area), or nonblocking (green area). (C) Heat map showing the competition matrix for 52 unique analytes (columns) against 233 unique ligands (rows)—with replicates removed—where each analyte/ligand pair is categorized as blocked (red), not blocked (green), intermediate (yellow), or self-blocking (dark red). White gaps represent missing/unaddressed permutations. See Supplementary Table S1 for full rendering of the heat map.

A premix assay format (Fig. 2A) was chosen for the epitope binning study because EBOV GP is a multivalent antigen comprising of a trimer of GP1 + GP2 heterodimers [22]. Figure 2B shows an overlay plot of the sensorgrams obtained for a ligand that gave clear binding responses in the premix binning assay, due to its facile regeneration, giving reproducible antigen binding responses, and full self-block. In this rapid, nonoptimized set-up, no attempt was made to optimize the concentration of the premixed antibodies and they were used in a ‘batch dilution’ mode as a 5-fold dilution of the supplied stock (corresponding to final antibody binding site concentrations of 53–1893 nM, with a mean of 507 nM). Clearly, this would not have achieved a molar excess of premixed antibody to antigen for some of the low-expressing antibodies, so we limited the premixed analytes to only those clones that showed clearly detectable ligand binding and above-mean expression (>20 μg from 1.2 mL culture). Since each analyte injection consumed 300 μL, we needed around 2 μg per EBOV GP per injection and since we had a limited supply of our purified antigen (0.5–1 mg), we would have nearly exhausted it if we injected EBOV GP premixed individually with all 321 antibodies (>300 injections, including antigen alone injections).

Figure 2C shows a heat map of the results from all active ligands (rows) and fully saturated premixes (columns) in the binning assay, revealing seven distinct epitope clusters or ‘communities’ without the inclusion of the benchmark antibodies. Due to the low expression of some clones and the requirement for their saturation of the antigen as premixed analyte, as judged by premixes giving a full block somewhere (self or elsewhere) in the competition matrix, the number of antibodies that were successful as premixes and gave clearly interpretable responses was rather low. Despite ligand attrition and the stringent requirement for premixed antibodies to achieve antigen saturation, which reduced our heat map to 52 analytes × 233 ligands, we were able in this nonoptimized ‘first pass’ binning to assign 234 antibodies to one of seven epitope communities without the use of benchmark antibodies **(**Supplementary Table S1, Figure S3**)**. HT-SPR binning heat maps including limited embedded standards, or only bidirectional ligand/analyte pairs were also generated **(**Supplementary Tables S2 and S3**)**.

### Antibodies representative of the epitope coverage of the full library were used as ‘pathfinder’ reagents to assign missing clones in a second pass, focused binning study

The results from our first pass binning led us to select a set of 15 ‘pathfinder’ antibodies that were representative of each epitope community and re-express them on a larger scale for use as high quality reagents that recapitulated the entire epitope diversity of the library in a few key clones. The pathfinders represented antibodies with good expression and performance in the binning assay as both analyte and ligand to facilitate their use in future binning experiments. We also scaled up the production of seven randomly selected clones from those that we had failed to assign to a community in our first pass binning, due to their poor performance as ligand, to see if we could assign these ‘unknowns’ in a more focused ‘second pass’ binning experiment. Also included were a set of 13 literature controls of known specificity from a collaborator that served as structural benchmarks to assess whether our SPR-derived epitope communities (represented by the aggregate coverage of our ‘pathfinders’) overlapped or extended beyond known epitopes.

**Figure 3:**
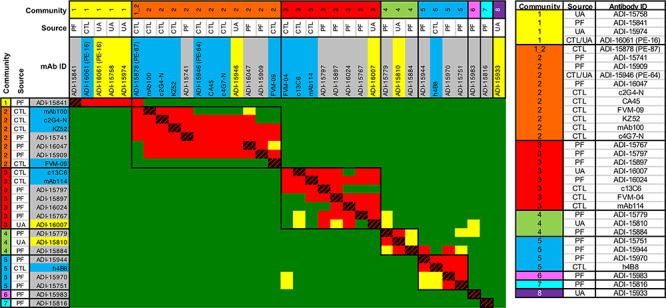
Second pass HT-SPR epitope binning (A) heat map and (B) tabulated assignments of the results from merging the pathfinders (PF), structural benchmarks from literature (CTL), and previously unassigned clones (UA) into a single focused binning experiment. All the structural benchmarks fell into our identified communities, allowing us to infer structural significance to our communities. For example, Comm1 was populated by control PE-16 (ADI-16061) known to bind to the viral stalk and Control PE-87 (ADI-15878) showed crosstalk of Comm1 + Comm2 and is located on the fusion loop [19]. Comm 2 was populated by base binding controls PE-64 (ADI-15946), c2G4-N, c4G7-N, CA45, FVM09, KZ52, and mAb100, all known to bind to the glycan cap and GP1 + GP2 interface, which consists of the internal fusion loop. Comm3 was populated by c13C6, FVM04 and mAb114, known to bind to the receptor binding site. Comm5 was populated by h4B8, known to bind to GP1 core. Comm4 and the rare communities (Comm6, 7, and 8) were not represented by these literature controls. For the aggregate epitope footprint of the structural benchmarks, see Fig. 6.

The pathfinders, unknowns, and structural benchmarks were merged into a single binning experiment that was performed in a completely independent manner than the first-pass binning. In this so-called ‘second-pass’ binning, conducted by a different operator working on a different LSA unit in a separate lab with a fresh batch of antigen and scaled-up antibodies, we successfully recapitulated the expected epitope coverage of the pathfinders **(**Supplementary Table S4**)** and used them to assign the 12 structural benchmarks to one of our seven identified communities as well as assign the unknowns to a community. Even with only the pathfinder antibodies, all seven epitopes were recapitulated (Supplementary Table S5). Consistent with our first pass results, most of the previously unassigned clones failed to perform as ligand, making us reliant on their performance in the role of premixed analyte to determine their epitope specificities. This was now made possible due to the larger yield available for those clones upon scale-up. The results from the second-pass binning are summarized as a heat map (Fig. 3A) and as list of assignments **(**Fig. 3B).

### SPR-derived epitope binning assignments closely match FACS-derived ones reported in the literature in an independent study

**Figure 4:**
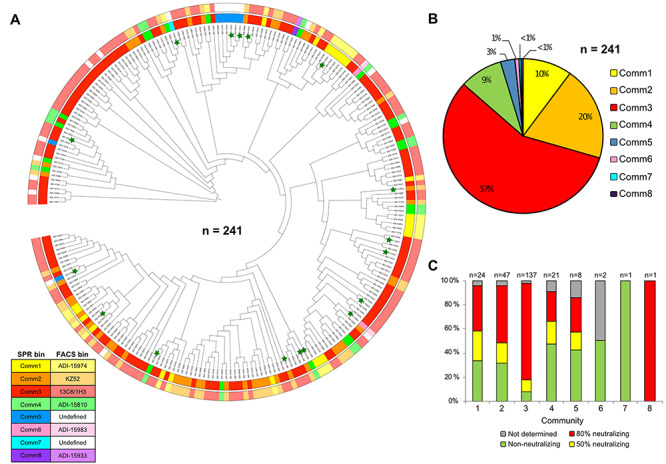
Benchmarking our HT-SPR epitope binning results against the literature. Antibody sequences, FACS binning and neutralization assay data were drawn from Bornholdt et al. [2]*.* (A) Sequence dendrogram of the antibody lineage of the variable heavy (VH) fragment alongside HT-SPR binning assignments (inner ring) and FACS binning assignments (outer ring) for the 241 antibodies that we empirically assigned to an epitope community. Antibody clones selected as pathfinders or unknowns to be tested in second pass binning are marked by a green star. Phylogenetic tree data generated by MUltiple Sequence Comparison by Log—Expectation (MUSCLE) [36], circular dendrogram figure constructed using Interactive Tree Of Life (iTOL) [37]. (B) Pie chart showing the distribution of 241 antibodies into the eight communities determined by HT-SPR. (C) Distribution of antibodies within each epitope community that reduced viral infectivity by 50, 80%, or determined to be nonneutralizing in a live virus plaque reduction neutralization assay. Neutralization assay was performed by and reported in Bornholdt et al. [2] as an endpoint titer based on the 50 and 80% thresholds in the number of plaques observed in control wells. The total number of antibodies within each assigned community is displayed at the top of each corresponding bar. Strongly neutralizing antibodies appear in all communities, except for Comm6 and Comm7, which were extremely rare.

Next, we compared our SPR binning results obtained from first and second pass experiments to literature assignments made by FACS [2]**.** Up to this point, antibody production had been blinded using internal clone names rather than ADI names, to test the reliability of our methods. Figure 4A shows a dendrogram of the antibody sequences for the 241 clones assigned to an epitope community by SPR. Clones are colored by their SPR-derived epitope community (inner circle) and compared to their FACS-derived epitope bin (outer circle). Antibody sequence does not appear to be fully predictive of epitope as unrelated sequence lineages converge upon the same epitope community **(**Fig. 4**,**Supplementary Fig. S6**)**, while highly related antibodies can bind to distinct, nonoverlapping epitopes, as shown by ADI-15731 (Comm3) and ADI-16052 (Comm6), both germline VH3–49 antibodies **(**Supplementary Table S1**)**.

Overall, we found excellent agreement between the SPR and FACS epitope binning determinations from independent studies, with only a few (4 out of 241) discordant assignments. We were able to align our communities with those determined by FACS as follows; ADI-15974 (Comm1), KZ52 (Comm2), 13C6/1H3 (Comm3), ADI-15810 (Comm4), ADI-15983 (Comm6), and ADI-15933 (Comm8). Comm5 and Comm7 were ‘undefined’ in the FACS binning. Community 3 appears to be immunodominant and accounts for over 50% of the assigned clones, consistent with the findings of Bornholdt et al. Communities 1, 2, 4, and 5 comprise the rest of the response with the exception of rare clones that belong to communities 6, 7 and 8. The emergence of the rare community 8, populated solely by ADI-15933 was not confirmed until the second-pass binning where antibody concentrations used for ligand and premixes were normalized across the test set, giving us more confidence that its epitope was truly unique, even though it failed to perform as ligand (Fig. 3). In the first-pass binning, ADI-15933 had failed to block any ligand (self or otherwise) as premixed analyte, so was excluded from our analysis because we could not rule out the possibility that its premixed concentration was sufficient to elicit a full block.

Ebola virus neutralization data and mouse survival data in a virus challenge experiment was obtained from Bornholdt et al**.** and merged with HT-SPR epitope binning assignments (Fig. 4C**,**Supplementary Tables S6 and S7). Strongly neutralizing antibodies were found in nearly all epitope communities except for the rare communities 6 and 7.

## DISCUSSION

### High-throughput epitope binning can be used to characterize hundreds of antibodies quickly with exquisite resolution

A key advantage of conducting epitope binning by HT-SPR is the universal applicability to test any panel of antibodies provided that the antigen is available in a purified, soluble form and a few micrograms of purified antibodies are available. Performing the binning assay does not require prior knowledge of the antigen structure [23]. Traditional epitope binning experiments rely on a ‘few-on-many’ approach where a subset of well-characterized control antibodies (‘knowns’) is used to probe the epitope coverage of a large antibody library (‘unknowns’). This limited scope approach compensates for standard analytical methods being low throughput, tedious, and often requiring prohibitively high sample consumption. The output of a ‘few-on-many’ approach lacks resolution because it is binary (blocking or nonblocking) relative to each standard and requires prior knowledge and availability of appropriate standards. In contrast, the use of HT-SPR expands the scope of the addressable competition matrix by enabling a deeper exploration of pairwise permutations in an unrestricted manner that clusters antibodies sharing similar blocking relationships and outputs high-resolution epitope bins or ‘communities’. Each epitope community is defined as a group of antibodies that share a similar, but not necessarily identical, blocking profile. This type of assignment is only possible when the number of antibodies being investigated is sufficiently large such as in a HT-SPR experiment. Epitope assignments are relativistic and can be performed without control or benchmark antibodies—communities are assigned in relation to the antibodies contained in the test set **(**Supplementary Table S1**)**. The high-throughput nature of the HT-SPR binning assay facilitates the identification of rare epitopes as antigen blockade is tested across so many antibodies that those showing unique blocking profiles are readily apparent. Of the 241 antibodies that we assigned to epitope communities, two or fewer were assigned to communities 6–8. Community 6 (ADI-15983 and ADI-16052) and community 8 (ADI-15933) were previously identified by FACS binning experiments, whereas community 7 (ADI-15816) was ‘undefined’ by FACS [2].

To obtain a clean result (block or not block) in a premix assay format, two caveats must be satisfied. First, the ligands must maintain their antigen-binding activity upon multiple rounds of regeneration. Second, the premixed antibody analyte must bind up and saturate (or nearly saturate) its recognized epitopes in the antigen sample to effectively diminish the antigen’s free concentration to a barely detectable level. A useful test to verify empirically that the premixed antibodies have achieved saturation of the antigen sample is a ‘self-block’ where the same antibody is used in the role of both analyte (premixed) and ligand (coupled to chip). Thus, a premixed antibody (analyte) that reduces antigen binding to a barely detectable level when probed by its coupled counterpart (as ligand) verifies that the premixed antibody is capable of saturating all epitopes in the antigen sample and therefore can be used reliably to assess the epitope-based competition of other ligands. Since we had no ‘a priori’ knowledge of the true binding affinities of any of the antibodies to our EBOV GP antigen (published kinetic data are avidity-influenced measurements on BLI [2]) we elected to use a large molar excess of each premixed antibody relative to the antigen concentration used, while balancing the need to conserve sample (both antigen and small-scale purified antibodies).

While we intended to explore the entire 321 × 321 ‘analyte × ligand’ competitive matrix, we observed about 25% ligand attrition due to ligands that showed poor antigen binding responses due to their low activity/affinity or being damaged (or conversely, unaffected) upon regeneration, so were excluded from our analysis. To conserve precious antigen, we elected to use as premixed analyte only those antibodies that had shown good ligand binding and good expression. Even if the antigen had not been precious, the yields of some antibodies in the small-scale expression would not have been sufficient to produce full saturation of the antigen, which reduced the number of premixed analytes that fulfilled this caveat. Conversely, epitope binning with a well behaved, monomeric antigen can be performed as a classical sandwich assay format which requires significantly less analyte because it does not depend on fully saturating the antigen. Unfortunately, for EBOV GP such a monomeric construct with biological relevance is not available.

### Epitope binning of large antibody sets reveals nuanced binding modes and provides a framework within which orthogonal data can be merged

**Figure 5:**
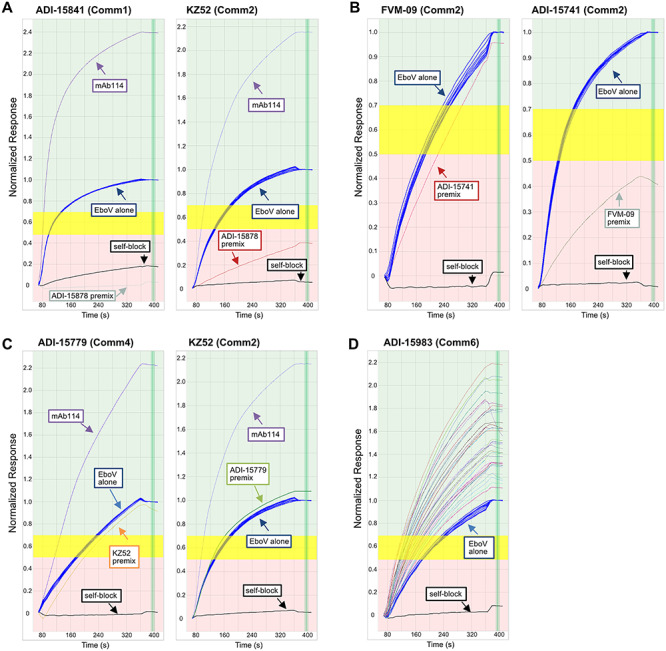
Examples of nuanced blocking behaviors observed by SPR, (A) blockade across two distinct epitope communities (cross-talking), (B) order-dependent blocking asymmetry, (C) possible antigen heterogeneity, and (D) rare epitopes. Each panel shows an overlay plot of sensorgrams, grouped by ligand (as named in the header), for the normalized binding responses of nine repeat injections of EBOV GP alone (blue) and the effect produced upon premixing EBOV GP with various antibodies (as analytes); the self-block is shown in black. **(A)** Premixed ADI-15878 (Comm1 + 2 cross-talker) significantly blocks coupled ADI-15841 (Comm1) and KZ52 (Comm2), while premixed mAb114 (Comm3) does not block antibodies in either community. (B) Premixed ADI-15741 (Comm2) does not block coupled FVM-09 (Comm2) but reversing the orientation produces blockade, suggesting possible allosteric modulation of EBOV GP by FVM-09 because FVM-09 showed this order-dependent blockade across most of the Comm2 antibodies. (C) Premixed ADI-15779 (Comm4) achieves a full self-block but produces barely any effect when presented to coupled KZ52 (Comm2), as observed by the premixed sample showing a very similar binding response as that of EBOV GP alone. The inverse is also true for premixed KZ52 on coupled ADI-15779, hinting at possible antigen heterogeneity where each antibody targets a separate antigen ‘subpopulation’. Conversely, premixed mAb114 (Comm3) gives a clear sandwiching response with both ADI-15779 and KZ52. (D) ADI-15983 (Comm6) is blocked only by itself (see self-block in black) and not by any other premixed antibodies tested (various colors) which all resulted in normalized binding responses >1.

Benchmark antibodies can add tremendous value to binning assays in providing ‘mapping’ information, since cross-blocking of such standards would infer overlapping epitopes. However, the binning assay itself is not reliant on them, but enhanced by them. Four interesting observations that emerged from our high throughput binning analysis that would have likely been overlooked in a simple ‘few-on-many’ approach are (i) cross-talking antibodies, (ii) asymmetric blockade, (iii) apparent antigen heterogeneity, and (iv) rare epitopes (Fig. 5).

While most of the antibodies fell neatly into one of seven discrete communities, a few antibodies were able to ‘crosstalk’ and block members of more than one community. An example of this behavior is ADI-15878, a structural benchmark clone**,** which blocked antibodies in Comm1 and Comm2 (Fig. 5A). This contrasts mAb114, a Comm3 member that only blocks other Comm3 members and shows significant sandwiching to antibodies from other communities. Most antibodies (like mAb114) in our study only blocked antibodies in their own community. In addition to ADI-15878, its genetic sibling, ADI-15742 also showed crosstalk of communities 1 and 2 (Supplementary Fig. S1). Structural data show that ADI-15878 and ADI-15742 bind to a ‘cryptic epitope’ within the viral fusion loop, which is believed to endow them with an ability to neutralize all five members of the Ebolavirus genus, Bundibugyo virus (BDBV), Sudan virus (SUDV), Marburg virus (MARV), Reston virus (RESTV), and Taï Forest virus (TAFV) [19,24]. In a ‘few-on-many’ binning paradigm, ADI-15878 (or ADI-15742) blocking is only known in context relative to the tested benchmarks. From our binning analysis, these two clones were clearly differentiated from the rest without examining their sequences—see Supplementary Table S2 showing first pass binning merged with a limited set of embedded controls.

When antibody competition is tested in both orders of addition, in rare cases, asymmetric or order-dependent competition is observed. In our study FVM-09 showed markedly asymmetric blockade of Comm2 members, only blocking them when presented first to the antigen (as premixed analyte), but not blocking any other antibody (except for itself) when presented second (as ligand) (Fig. 5B). Indeed, FVM-09 has been reported to act cooperatively in cocktails due to it possibly triggering an ‘induced’ epitope, an epitope that is either formed or exposed upon binding of GP by another antibody [7]. Therefore, high throughput binning can reveal possible cases of allosteric modulation that may enable nuanced mechanisms of action (Supplementary Table S2).

Visual inspection of the SPR sensorgrams suggests the presence of antigen heterogeneity. Comm4 and Comm5 antibodies appeared to bind a subpopulation of the antigen that was distinct from that recognized by other communities. This manifests in the sensorgrams as premixes of Comm4 and Comm5 antibodies showing ‘no effect’ on the binding of some ligands, while completely blocking others [25]**.** An example is shown in Fig. 5C where ADI-15779 (Comm4) and KZ52 (Comm2) fully self-block but appear to have no effect on EboV binding to the opposing antibody when premixed in solution**.** The antigen appeared conformationally intact as judged by its high degree of homogeneity when tested by standard analytical sizing methods (Supplementary Fig. S1), so the existence of functional subpopulations that are differentially recognized by antibodies in the panel would not have been obvious otherwise. Despite possible antigen heterogeneity, each antibody still exhibits reliable binding to EBOV GP, full self-block and thus did not affect our ability to assign antibodies to epitope communities.

**Figure 6:**
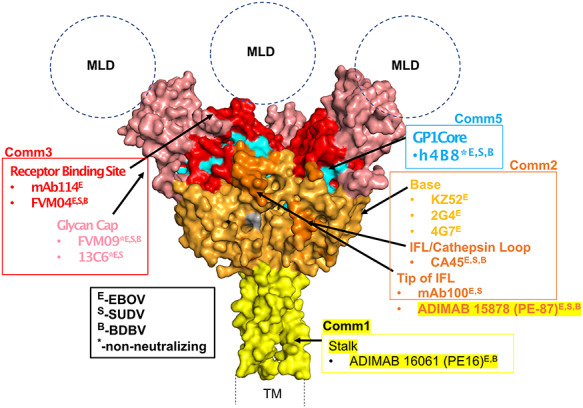
Crystal structure of the prefusion trimeric EBOV GP (lacking the mucin-like (MLD) and transmembrane (TM) domains [38]. Structural epitopes are color-coded in the surface representation of EBOV GP, and benchmark antibodies along with their communities have been assigned and highlighted for the various epitopes. Additionally, neutralizing or nonneutralizing properties of the different antibodies have been shown in superscript as described in the legend (black box). Communities 4, 6, 7, and 8 are not highlighted due to their novelty and lack of similarities within any of the benchmark antibodies used in this study.

High-throughput epitope binning of large panels of antibodies is uniquely positioned to identify rare epitopes. As the blockade matrix size increases, these rare communities are repeatedly assayed and confirmed to only block within their small communities (Comm5 and Comm6) or only to self-block (Comm7) **(**Fig. 5D). Mapping the epitope footprint of structural benchmarks show that epitope communities belonging to large, exposed epitopes generate large numbers of community members (Comm3) while smaller number of antibodies bind to more occluded epitopes such as the GP1 core (Comm5) (Fig. 6). Assembling antibody cocktails inclusive of these rare epitopes allows further probing of antibody combinations to uncover synergistic effects and avoid biasing therapies to an immunodominant epitope that may be cleaved as a decoy or be tolerant of high rates of mutation.

In our binning study, in addition to the trimeric EBOV GP we also tested a cleaved form of GP (GPcl), the product of cathepsin cleavage in the endosome and loss of glycan cap. We observed that antibodies in communities 1–3 bound to GPcl, while communities 4–8 did not, suggesting they targeted the sequence that was cleaved away from the viral membrane (Supplementary Table S8). One notable exception was FVM-09 (Comm2) which did not bind GPcl, consistent with its known epitope specificity [26].

### Antibody cocktails that comprise antibodies targeting disparate nonoverlapping epitopes enhance neutralization potency by combining multiple mechanisms of action.

Cocktails that combine several antibodies targeting disparate epitopes can unlock unexpected synergistic effects that enhance neutralization activity beyond the additive contribution of each antibody, as has been demonstrated in treatments for Botulinum neurotoxins [10], Sudan virus [27], Ebola virus [28], and HIV-1 [29]. Furthermore, antibodies that individually do not display neutralizing activity as a monotherapy may exert a synergistic neutralizing effect when combined in a cocktail [7]. Understanding the epitope landscape of antibodies generated by the human immune response to authentic viral infection is necessary to rationally assemble therapeutic antibody cocktails that condense the entire immune response to a handful of key clones that confer protection. However, the human immune system responds differently to different pathogens. Previous reports show that neutralizing antibodies produced in response to *Staphylococcus aureus*, a commensal pathogen, are strongly germline-biased [30]. A recent study of the human immune response to vaccination by Yellow Fever Virus 17D also reported a germline-encoded neutralization [31]. Conversely, by merging our empirically determined epitope communities with functional data from the literature, we found that anti-EBOV GP antibodies from multiple epitope communities were able to neutralize in a cell-based assay (Fig. 4C) and confer protection in a mouse challenge model (Supplementary Table S7**)**. These findings reinforce the need to evaluate the epitope landscape of the human immune repertoire to each pathogen empirically.

While antibody affinity and developability can be engineered, the binding epitope of an antibody is an innate property that cannot be engineered downstream. In traditional antibody discovery workflows, high-throughput sequence and functional screens are often performed upstream while epitope bins are only assigned downstream for a limited set of lead candidates. Additionally, *in vitro* neutralization does not always correlate with *in vivo* protection [28]**.** Triaging antibodies based on their epitope specificity rather than their neutralization ensures the broadest epitope coverage of the antigenic surface, which can potentially activate multiple mechanisms of action in concert, minimize mutagenic escape, as well as leverage unexpected cooperative or synergistic mechanisms that may exist across antibodies [11, 32].

Designing effective and long-lasting therapies to combat viral infections is confounded by the innate ability of viruses to rapidly mutate and evade their host immune responses by producing so-called ‘escape mutant’ variants. Human influenza has been shown to escape most antibodies with single mutations and prioritizing antibodies only by their ability to neutralize virus, without any epitope binning context, may skew coverage to an immunodominant epitope that can be readily rendered ineffective with a single point mutation [12]. Exposure to strongly neutralizing antibody monotherapies can drive the emergence of Ebola virus escape mutations in nonhuman primates [11]. Potential escape mutations that disrupt binding of KZ52 (Comm2), mAb100 (Comm2), and mAb114 (Comm3) without affecting EBOV GP folding have also been discovered [32]. Another evolutionary strategy employed by viruses to evade a host's immune response is via the generation of immunodominant ‘decoy epitopes’ on soluble forms of their glycoproteins that subvert the immune response toward production of nonneutralizing antibodies. This escape mechanism is present in Ebola virus in the form of secreted glycoprotein isoform (sGP) [33]. Cocktails that carefully combine antibodies targeting disparate epitopes by utilizing knowledge of the epitope landscape early in the drug discovery process, as enabled by high throughput binning, may be better equipped to overcome mutagenic escape and decoy epitopes.

Fully characterizing the epitope distribution of antibodies generated by the human immune response to authentic infection viral infection aids in the understanding of the features of antibodies that ultimately confer *in vivo* protection. The benefits of epitope binning large panels of antibodies are agnostic to instrumentation and can be performed on any label-free platform with varying efficiencies in time, sample consumption, and practical convenience. Additionally, the rational design of antibody cocktails can be applied beyond treating infectious disease. For example, in MET-amplified tumor models, antibodies that bind to nonoverlapping epitopes of receptor tyrosine kinase MET enhanced antagonistic activity [34], while a combination of two anti-EGFR antibodies addressing disparate epitopes helped circumvent cetuximab resistance in metastatic colorectal cancer models [35]. Screening for appropriate epitopes early in the discovery process enables cocktail therapeutics to take advantage of cooperativity, lower the risk of mutagenic escape, and improve long-lasting function whether the goal is neutralizing viral infection or treating malignant tumors.

## Supplementary Material

Supplemental_tbaa016

Supplemental_Tables_5_1_tbaa016
