# Supplementary material for: Rapid exploration of the epitope coverage produced by an Ebola survivor to guide the discovery of therapeutic antibody cocktails
Source: Antib Ther. 2020 Aug 1;3(3):167–78. doi: 10.1093/abt/tbaa016 (PMC7454256; doi:10.1093/abt/tbaa016)
Supplement: Supplemental_tbaa016 [file supplemental_tbaa016.docx]

**SUPPLEMENTAL FIGURES**


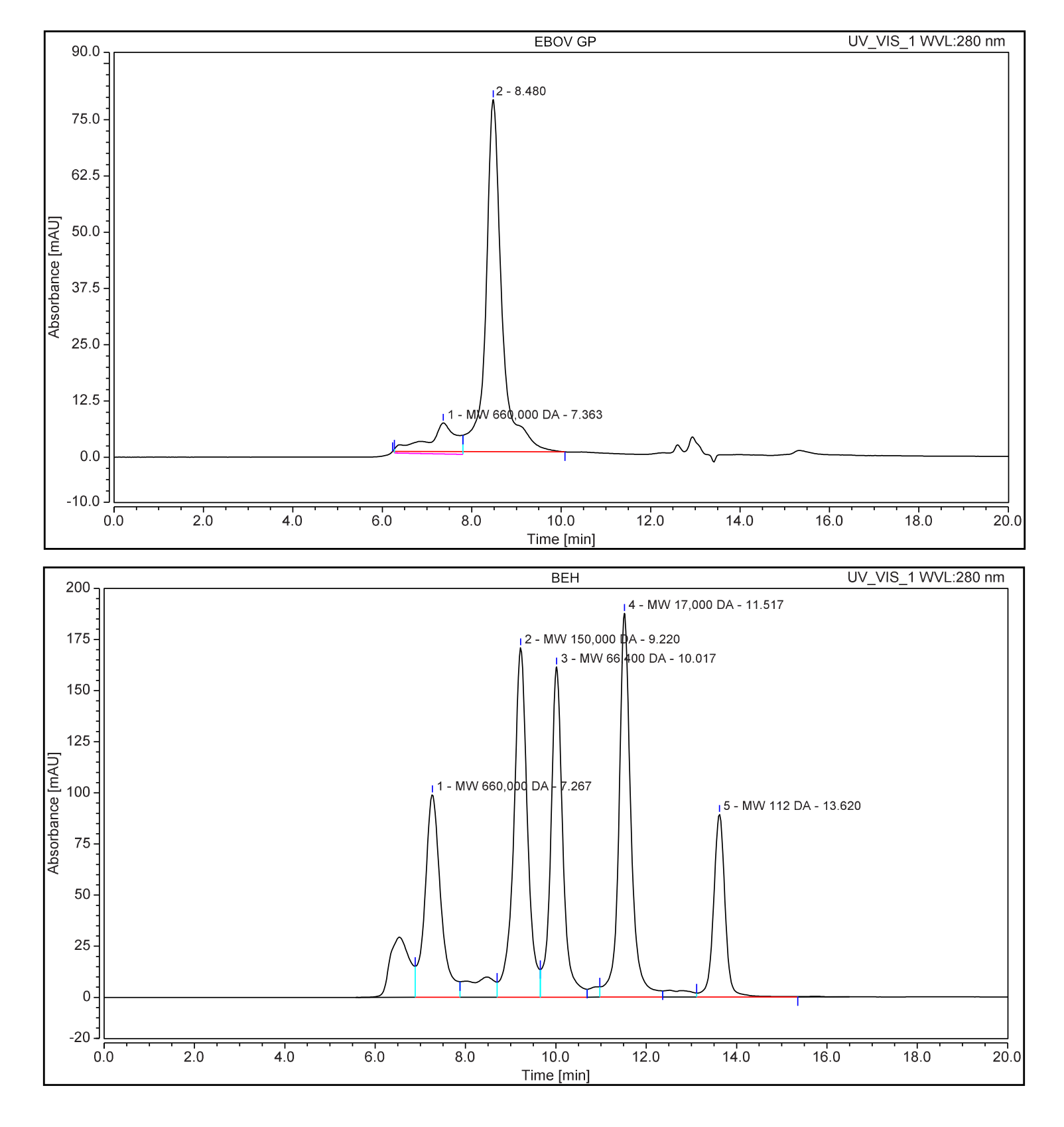


**Supplemental Figure S1.** Analytical size exclusion chromatography trace of EBOV GP sample (a trimer of GP1+GP2 heterodimer with a theoretical assembled total molecular mass of 207 kDa) used in epitope binning experiments (upper chromatogram). Sample was injected into MAbPac SEC-1 4x300mm column in 50 mM sodium phosphate, 300 mM sodium chloride pH 6.8 on a Thermo Scientific UltiMate 3000 HPLC system. The glycosylated EBOV GP migrated at ~386 kDa based on BEH200 molecular weight standards as shown in the lower chromatogram (Waters).


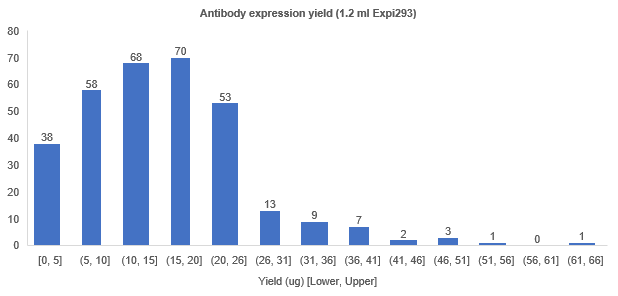


**Supplemental Figure S2.** Distribution of purified antibody yields (μg) from small scale 1.2 ml Expi293 transient expression. The majority of clones were expressed at over 5 μg which yielded sufficient protein to conduct the ‘first pass’ epitope bin, using all clones as ligand and a smaller, curated set for use as analyte (premix), which necessitated the use of a high enough concentration to saturate the antigen.

**Supplemental Figure S3**. Epitope community assignment distribution among the VH germlines used in the panel of 241 anti-EBOV GP antibodies that were assigned to an epitope community.

**SUPPLEMENTAL TABLE CAPTIONS**

**Supplemental Table S1.** Heat map of 52 analytes (columns) x 233 ligands (rows) resulting from the first pass HT-SPR epitope binning experiment without the use of any benchmark antibodies. Numbers in the heat map represent the normalized binding response of that premixed sample relative to EBOV GP alone (normalized to 1) per ligand Heat map is colored by premixed analytes that show sandwiching (green), intermediate blocking (yellow), and full blocking (red). Seven distinct epitope communities emerge (numbered 1-7). Clones that are assigned to two bins (e.g. 1_2, 4_5) show ‘crosstalk’ or blockade across two communities. Within each community, clones are roughly sorted by their VH germline (shown) to show both, the capture of clonal families and the convergence of unrelated VH germlines per epitope community. This Table is a detailed rendering of the one shown in **Figure 2C**.

**Supplemental Table S2.** Epitope binning assignments as in Supplemental Table S1 but with the addition of merged epitope binning assignments from a limited set of benchmark antibodies.

**Supplemental Table S3**. Epitope binning assignments as in Supplemental Table S2 showing only full bidirectional ligand-analyte pairs by removing analyte-only antibody premixes.

**Supplemental Table S4.** Heat map of 35 analytes (columns) x 24 ligands (rows) from the second pass HT-SPR epitope binning experiment selected from pathfinder antibodies, benchmark antibodies, and antibodies with unresolved epitope bins from the first HT-SPR pass. Numbers within heat map represent the normalized binding response of that premixed sample relative to EBOV GP alone (normalized to 1) per ligand. As before, heat map is colored by premixed analytes that show sandwiching (green, intermediate blocking (yellow), and full blocking (red). The seven distinct epitope bins that emerge are now assigned with benchmark antibodies along with an eighth epitope bin that is identified by the analyte only ADI-15933 premix run.

**Supplemental Table S5.** Heat map of 15 Pathfinders (used as both ligand and analyte) distilled from the results of the second pass HT-SPR epitope binning experiment, showcasing full representation of the seven epitopes identified in the first pass binning with a significantly reduced subset of clones.

**Supplemental Table S6.** List of anti-EBOV GP antibodies with epitope binning assignments from merged first pass and second pass HT-SPR assay compared to small scale expression yield and FACS binning and neutralization activity as reported in *Bornholdt et al.*^2^. Out of 241 total assigned antibodies, only 4 conflicts appear in epitope assignments. We assigned a total of 8 bins, identical to the FACS dataset aside from two clones in the ‘mucin-like’ epitope. (+) = neutralizing, (-) = non-neutralizing, ND = not defined, NT = not tested, (x) = undefined epitope, colored boxes denote epitope assignment.

**Supplemental Table S7.** List of anti-EBOV GP antibodies with epitope binning assignments from merged first pass and second pass HT-SPR assay compared Ebolavirus challenge in a mouse survival model as reported in *Bornholdt et al.*^2^.

**Supplemental Table S8.** Heat map generated with GPcl as antigen using small-scale antibody preparations and a limited set of benchmark antibodies. Notably, only antibodies in Communities 1-3 bound GPcl, while those in Communities 4-8 did not. Of the benchmarks tested, FVM09 showed no binding to GPcl, despite it being assigned to Community 2 when using the trimeric GP construct.

**SUPPLEMENTAL MATERIALS AND METHODS**

**HT-SPR epitope binning**

Epitope binning was performed in a premix assay format on Carterra’s LSA SPR instrument equipped with HC30M chip type at 25ºC and in a run buffer of HBS-TE (10 mM HEPES pH 7.4, 150 mM NaCl, 3 mM EDTA, 0.05% Tween-20) supplemented with 0.5 mg/ml BSA. The LSA uses an automated choreography between two microfluidic modules, a 96-channel print-head (96PH) and a single flow cell (SFC), to deliver samples to the sensor chip in different ways. Surface preparation involved priming the entire system (96PH and SFC) in a run buffer of 50 mM MES pH 5.5 with 0.05% Tween-20, activating the chip with a freshly prepared solution of 0.4 M 1-ethyl-3-(3-dimethylaminopropyl)carbodiimide (EDC) + 0.1 M N-hydroxysulfosuccinimide (NHS) + 0.1 M MES pH 5.5 in the SFC, printing the antibodies (diluted into 10 mM sodium acetate pH 4.5) via in the 96PH and quenching unreactive esters with 1 M ethanolamine.HCl pH 8.5 in the SFC. Activation (in the SFC), coupling (in the 96PH), and quenching (in the SFC) steps were each allowed 7 min. For the “first pass” binning, 321 antibodies expressed as 1.2 ml cultures (small-scale) were “batch diluted” 50-fold from their supplied stocks and printed as a 384-ligand array by docking the 96PH sequentially onto different print block locations. To fill out the entire capacity of the 384-array, some antibodies were printed onto two or more spots (to provide internal replicates) and a limited number of controls (a subset of the structural benchmarks) coupled at 2-5 µg/ml were included as embedded standards. In the “second pass” binning, a smaller set of 24 antibodies comprising Pathfinders and Unknowns expressed as 30 ml cultures (large-scale), and a full set of controls (the structural benchmarks) were normalized to 5 µg/ml and coupled as a 96-ligand array.

Binning was performed in the SFC, primed in a run buffer of HBS-TE + BSA. Immediately after preparing the surfaces for use in the first-pass binning, an initial binding test and regeneration scouting experiment was performed to find appropriate conditions for antigen binding. Most ligands appeared to give robust and reproducible binding responses when screened at 80 nM EBOV GP binding sites (assuming 69 kDa per binding site) and regenerated with 75 mM phosphoric acid. Ligands showing good responses and good expression yields were prioritized for use as premixed analyte in the first-pass binning and “batch diluted” 5fold from their original supplied stocks to prepare the “premixed samples” of 80 nM EBOV GP + antibody (as analytes), intentionally with the premixed antibody at molar excess (in binding sites). Samples were allowed to reach equilibrium prior to injecting them for 5 min. Multiple samples of EBOV GP alone were injected periodically throughout the assay to provide an average “antigen only” response used for threshold setting in the analysis. In the second pass binning, all antibodies were used in the role of both ligand and analyte, and premixes were prepared using 50 nM EBOV + 300 nM antibody (binding sites).

Data were processed and analyzed in Carterra’s Epitope Tool software. Premixes giving binding responses <50% of the EBOV GP alone samples were assigned as “blocked” whereas those above this value were “not blocked”. Responses falling between 50-70% blocked were assigned as “intermediate”. These global settings were adjusted manually (per ligand) at the discretion of the analyst. Competition results were visualized as a heat map where red, yellow, and green cells represent blocked, intermediate and not blocked analyte/ligand pairs, respectively. Data from separate “first pass” experiments were merged into a single heat map that was sorted manually, giving rise to white cells representing unaddressed permutations. Ligands showing poor performance (inactive, barely binding or failure to regenerate) were excluded. Premixed analytes showing no blockade of any ligand were also excluded from the first-pass binning.
